# Prevalence of extended-spectrum β-lactamase (ESBL) and molecular detection of *bla*TEM, *bla*SHV and *bla*CTX-M genotypes among *Enterobacteriaceae* isolates from patients in Khartoum, Sudan

**DOI:** 10.11604/pamj.2020.37.213.24988

**Published:** 2020-11-03

**Authors:** Maha Hassan Dirar, Naser Eldin Bilal, Mutasim Elhadi Ibrahim, Mohamed Elamin Hamid

**Affiliations:** 1 College of Medical Laboratory Sciences, University of Khartoum, Khartoum, Sudan,; 2 Department of Basic Medical Sciences, Microbiology Unit, College of Medicine, University of Bisha, Bisha, Kingdom of Saudi Arabia,; 3 Department of Clinical Microbiology and Parasitology, College of Medicine, King Khalid University, Abha, Kingdom of Saudi Arabia

**Keywords:** Antimicrobial resistance, genotyping, Gram negative bacteria, *Klebsiella pneumoniae*, *Escherichia coli*

## Abstract

**Introduction:**

the emergence of antibiotic resistance pathogens is an important health risk. Usually Gram negative bacteria acquire resistance to beta-lactam antibiotics by beta-lactamase production. The objectives of this study was to assess the prevalence of ESBL and to detect the frequency of blaTEM, blaSHV and blaCTX-M genotypes among ESBL producing Enterobacteriaceae isolates from patients in Khartoum, Sudan.

**Methods:**

a total of 171 isolates of Enterobacteriaceae were recovered from hospitals in Khartoum, Sudan (2014 -2015) were used to detect ESBL production using disc diffusion method. blaTEM, blaSHV and blaCTX-M genes were investigated by PCR based methods using gene-specific primers.

**Results:**

the high resistance among Enterobacteriaceae was noticed in ciprofloxacin (72%) and ofloxacin (73%). ESBL production was mainly in Escherichia Coli (38%) and Klebsiella pneumonia (34%). Prevalent genotypes were blaTEM (86%), blaCTX-M (78%) and blaSHV (28%). These were found mainly in Escherichia Coli (38%, 37%, 2%) and K. pneumonia (34%, 31%, 26.1%). The majority of ESBL producing isolates possess more than one ESBL genes.

**Conclusion:**

the ESBL production in Enterobacteriaceae was high, with blaTEM and blaCTX-M genotypes more prevalent. Public health and laboratory standard of excellence is needed to reducing the spread of resistant pathogens.

## Introduction

The rapid development and spread of antibiotic resistance in health care facilities is an increasingly worrying public health trend. A known mechanism by which Gram-negative bacteria acquire resistance to beta-lactam antibiotics is the production of beta-lactamase enzymes [1, 2]. The extended-spectrum β-lactamase (ESBL) are plasmid-mediated enzymes that are able to hydrolyze and inactivate broad spectrum β-Lactams antimicrobials namely: third-generation cephalosporins, penicillins and aztreonam but are inhibited by clavulanic acid [3]. Extended spectrum beta-lactamases (ESBLs) are commonly found in *Klebsiella* species and *E. coli*, but have been described in other members of the *Enterobacteriaceae* such as *Enterobacter, Serratia, Citrobacter, Proteus* and *Salmonella* [4]. These organisms are causal agents of different infections such as urinary tract infection, septicemia, hospital-acquired pneumonia, intra-abdominal abscess, brain abscess and device-related infections [5]. ESBLs are now a problem in hospitalized patients worldwide.

The ESBL phenomenon began in Western Europe, most likely because the expanded-spectrum-lactam antibiotics were first used. However, it did not take long before ESBLs had been detected in the United States and Asia. The prevalence of ESBL production among members of the *Enterobacteriaceae* differs very much from country to country. The worldwide prevalence of ESBL-producing *Enterobacteriaceae* has increased over time. Evidence of ESBL-producing *Enterobacteriaceae* can be found in all regions of the world. Studies have indicated high levels of the ESBL phenotype in Asia, predominantly in *Klebsiella* strains mostly from China, Korea, Japan and India [6, 7]. Studies conducted in Africa mainly focused on the northern and eastern parts of the continent, while only rare studies were carried out in the rest of the continent [8]. In Nigeria, phenotypic ESBL production test demonstrated that 65% of the isolates were ESBL producers [9]. In Sudan, there are few data available regarding the incidence of ESBL producing microorganisms. These studies have revealed high rate of β-Lactamase production and high resistance level for 3^rd^ generation cephalosporin was noticed [10-12]. The objectives of this study were to assess the prevalence of ESBL and to detect the frequency of *bla*TEM, *bla*SHV and *bla*CTX-M genotypes among ESBL producing clinical *Enterobacteriaceae* isolates from patients in Khartoum, Sudan.

## Methods

**Ethical approval:** this study was approved by the Ethic Committees of the Ministry of Health, Khartoum State, Sudan.

**Study design and setting:** a cross sectional and descriptive analytical study was carried out between May 2014 and February 2015. The research received materials from five referral hospitals in the state, namely: Khartoum Teaching Hospital, Bahri Teaching Hospital, Soba University Hospital, Omdurman Teaching Hospital and Sharg Alnil Hospital.

**Sample size and study population:** specimens (n = 171 isolates) were collected from infected patients who attended the above mentioned five hospitals. The isolates were recovered from a total sample size of 231 clinical specimens. The sample size was calculated according to the following equation:

$$\text{n=}\frac{NZ^{2}pq}{(N-1)d^{2}+z^{2}pq}$$

Where: n, sample size; N, total target population, Z, area under the normal curve corresponding to 95%confidence level =1.96 (P=0.5); q, (1-p) = 0.5 and d, desire margin error [13].

**Specimen collection and transportation:** the specimens were collected from different clinical specimens in the five referral hospitals in Khartoum and immediately transported to the laboratory for analysis. The strains were isolated and identified in the microbiology laboratories of the College of Medical Laboratory Sciences, University of Khartoum, Khartoum, Sudan following standard methods [14]. Then, BD Phoenix system (Becton Dickinson, ABD) was used to confirm the identification of the 171 isolates as per company instructions.

**Screening of ESBL production:** detection of and phenotypic confirmation of ESBL production were carried out using double-disk synergy test and E-test.

**Disc diffusion method:** anti-bacterial susceptibility testing of the isolates was performed by the Kirby-Bauer disk diffusion assay on Mueller-Hinton agar medium against 14 antibacterial agent disks following the Clinical Laboratory Standard Institute (CLSI, 2011) guidelines [15]. The antibacterial agents tested include: amikacin (30 μg), amoxicillin (10 μg), amoxicillin-clavulanic acid (30 μg), ceftazidime (30 μg), ceftriaxone (30 μg), cefotaxime (30 μg), cefuroxime (30 μg), ciprofloxacin (5 μg), gentamicin (10 μg), nitrofurantoin (50 μg), ofloxacin (5 μg), and trimethoprim-sulfamethoxazole (25 μg) Cefoxitin (30 μg), Imipenem (10μg) (Oxoid, England).

**Double-disk synergy test:** the double-disk synergy test was performed as described in Jarlier *et al*. [16]. This test was carried out immediately along with susceptibility testing of each isolate. A susceptibility disk containing amoxicillin-clavulanic acid was placed in the centre of the plate, and disks containing ceftazidime and cefotaxime were placed 30 mm (centre to centre) from the amoxicillin-clavulanic acid disk. A clear extension of the edge of the inhibition zone of cephalosporin towards amoxicillin-clavulanic acid disk is interpreted as positive for ESBL production.

**E-test:** the E-test ESBL strips included ceftazidime/ceftazidime + clavulanic acid and cefotaxime/cefotaxime + clavulanic acid, for which the recommended ratio value indicates the presence of an ESBL as described by Vercauteren *et al*. [17]. The E-test procedure, reading, and interpretation were performed according to the manufacturer´s instructions (Liofilchem s.r.l. Italy). In brief, isolated colonies were suspended in saline (0.85% NaCl) to achieve an inoculum equivalent to 0.5 McFarland standards. A swab was soaked in the suspension and inoculated on a Mueller-Hinton agar plate and allowed to dry completely. An ESBL E test strip was applied on the agar surface with sterile forceps and the plate was incubated at 37°C overnight. ESBL results were read either as MIC CTX ≥ 0.5 and CTX/CTL ratio ≥ 8 or CAZ ≥ 1 and CAZ/CAL ratio ≥ 8 is indicative of ESBL production. Deformation of ellipses or the presence of a “phantom zone” is also indicative of ESBL production.

**Molecular detection of b-lactamase-encoding genes:** ESBL-producing strains were examined for the presence of *bla*TEM, *bla*SHV and *bla*CTX-M genes by PCR methods using gene-specific primers. Deoxyribonucleic acid (DNA) was extracted from ESBL producer strains using the Wizard Genomic DNA Purification Kit according to company instruction (Promega, USA). Oligonucleotide primers used to detect b-lactamase-encoding genes were as follows: *bla*TEM (931 bp): F-TCCGCTCATGAGACAATAACC and R-TGGTCTGACAGTTACCAATGC [18], *bla*SHV (868 bp): F-TGGTTATGCGTTATATTCGCC and R- GGTTAGCGTTGCCAGTGCT [19] and *bla*CTX-M (909 bp): F-TCTTCCAGAATAAGGAATCCC and R-CCGTTTCCGCTATTACAAAC [18]. The extracted DNA was examined for the presence of *bla*TEM, *bla*SHV and *bla*CTX-M encoding ESBL producing organisms. The PCR amplification was carried out using GoTaqq PCR Master Mix (Promega) with total reaction volume of 25μl. Each reaction mixture contains 3μl of DNA template, 1.5μl of each primer, 12.5μl of Master Mix reagent and 6.5μl of RNase-free water.

Amplification was performed on the SensoQuest Lab Cycler (Goettingen, Germany) with the following cycling parameters of denaturation, annealing, and extension adjusted according to the gene type. For *bla*TEM the parameters were started by 95°C for 5 minute followed by 35 cycles of 95°C for 1 minute 58°C for 1 minute 72°C for 1 minute final extension at 72°C for 5 minutes, *bla*SHV the parameters were started by 95°C for 5 minute followed by 35 cycles of 95°C for 1 minute 60°C for 30 second 72°C for 1 minute final extension at 72°C for 5 minutes and *bla*CTX-M also started by 95°C for 5 minute followed by 35 cycles of 95°C for 1 minute 58°C for 1 minute 72°C for 1 minute and final extension at 72°C for 5 minutes. All negative and positive controls were prepared in the same manner as the DNA templates used for the clinical isolate. The amplification products were detected by gel electrophoresis, 7μl amplified product of each reaction was loaded on 1.2% agarose gel containing ethidium bromide (1μg/ml)and electrophoresis in 80 volt for 30 minutes. The DNA ladder marker (with size 10.0 KB) was used as a standard molecular weight (MW) to determine the size of PCR products. After electrophoresis, PCR products were visualized under ultra violet illumination in capture by digital image analysis. The following control organisms were used during isolation, identification and molecular analysis of ESBL microorganisms: *Escherichia coli* (C600), *E. coli* (ATCC 25922), *Staphylococcus aureus* (ATCC 25923) and *Klebsiella pneumonia* (ATCC 700603).

## Results

A total of 171 members of *Enterobacteriaceae* and related gram negative bacteria were collected from patients at five hospitals in Khartoum state, Sudan, for the detection and characterization of extended spectrum β-lactamase (ESBL) producers.

**Distribution of clinical isolates according patient and hospital criteria:** of the 171 patients, 92(53.8%) were females and 79 (46.2%) were males. The majority of the patients were in the age group more than 66 years (23%) followed by 32-49 years (21%), less than 7 years(14%), 24-31 years (14 %), 58-65 years (13%), 50-57 years (12.0%),8-15 years (1 %) and 16-23 years (0.6%). Most of clinical isolates were isolated from Soba University Hospital and Sharg Elnil Hospital (66 isolates for each), followed by Bahri Hospital (20 isolates) and Khartoum Teaching Hospital (16 isolates). The type and distribution of clinical isolates recovered from hospitals according to departments is shown in Table 1. Of the 171 pathogens, 85% were nosocomial infection isolates whereas 15% were derived from community acquired infection.

**Table 1:** distribution of clinical isolates according to hospital departments and the type of infection

Hospital department	Number (%) of nosocomial infection isolates	Number (%) of community acquired infection isolates
Internal medicine	13 (8%)	8 (5%)
ICU	19 (11%)	0 (0.0%)
Pediatric	1 (0.6%)	5 (3.0%)
Surgery	111 (65%)	4 (2.0%)
Nephrology	1 (0.6%)	7 (4.0%)
Kwashiorkor	1 (0.6%)	0 (0.0%)
ENT	0 (0.0%)	1 (0.6%)
**Total**	**146 (85%)**	**25 (15%)**

**Antimicrobial susceptibility testing:** the resistance rates of the 171 *Enterobacteriaceae* and other gram negative isolates were as follows: amikacin, 12.3%; amoxicillin, 98.8%; amoxicillin + clavulanic acid, 95.9%; cefotaxime, 91.8%; cefoxitin, 45.0; ceftazidime, 91.8; ceftriaxone, 91.8; Cefuroxime, 96.5; ciprofloxacin, 71.9; gentamicin, 45.0; Imipenem, 22.8; nitrofurantoin, 45.6 ofloxacin, 72.5 and trimethoprim sulphamethoxazole, 77.8.

**Figure 1:**
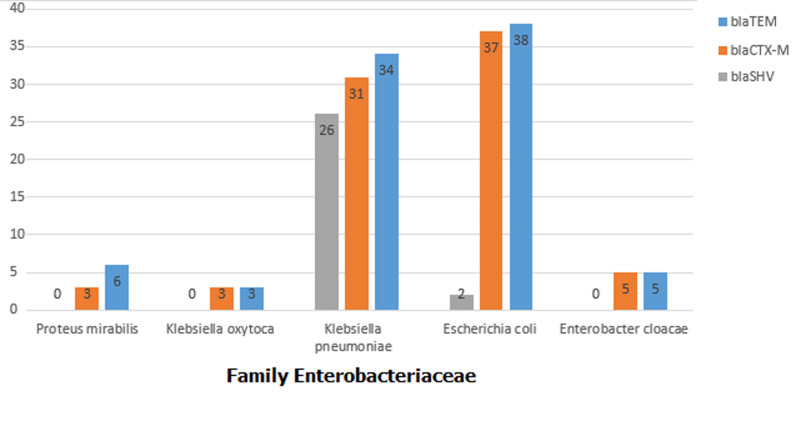
detection of *bla*TEM, *bla*SHV and *bla*CTX-M genotypes in clinical isolates of the family *Enterobacteriaceae* from patients in Khartoum, Sudan (2014-2015)

**Screening for ESBL production:** ESBL production detected among the 171 *Enterobacteriaceae* isolates was as follows: *E. coli*, 38%; *Klebsiella pneumonia*, 34%; Proteus mirabilis, 6%; 5%, Enterobacter cloacae and 3% in *Klebsiella oxytoca* (Figure 1).

**Figure 2:**
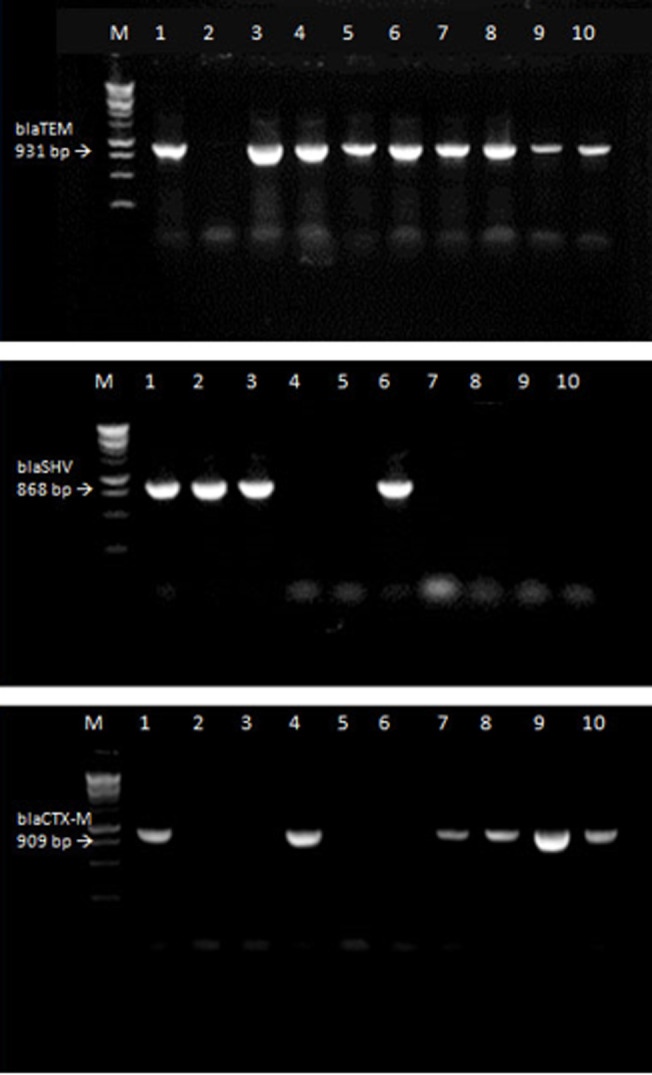
PCR assays for the detection of genotypes from phenotypically ESBL positive isolates; *bla*TEM (931 bp) in the upper gel: lanes 1, 3-10 were *bla*TEM positive isolates and lane 2, negative control isolate; *bla*SHV (868 bp) in the middle gel: lanes 1, 2, 3 and 6 were *bla*SHV positive isolates; lanes 5, 7-10 were *bla*SHV negative isolates and lane 4, a negative control isolate; *bla*CTX-M (909 bp) in the lower gel: lanes 1, 4, 7-10 were *bla*CTX-M positive isolates; lanes 3, 5, 6 were *bla*CTX-M negative isolates and lane 2, negative control isolate; Lane M represents the molecular weight marker (1 kb DNA Ladder, Promega)

**Molecular detection of TEM, CTX.M and SHV genes:** PCR assays for the detection of genotypes from phenotypically ESBL positive isolates among the major *Enterobacteriaceae* clinical isolates from patients in Khartoum, Sudan (2014-2015) is shown in Table 2 and Figure 2. Major genotypes in the clinical isolates from patients in Khartoum is shown in Table 2. The most prevalent genotype was found to be *bla*TEM (86%) followed by *bla*CTX-M (78%) then *bla*SHV (28%). *bla*TEM, *bla*CTX-M and *bla*SHV genes were found mainly in *E. coli* (38%, 37%, 2%) and *K. pneumonia* (34%, 31%, 26.1%). The majority of ESBL producing isolates possess more than one ESBL genes. 86% of the examined 65 isolated were positive for TEM gene which is the most common. This is followed by CTX-M (78%) then SHV gene (28%).These genes were distributed in all of the five hospitals, but the majority were from Soba University Hospital (55.2%) followed by Sharg Elnil Hospital (30.4%), Khartoum Teaching Hospital (7.2%), Bahri Teaching Hospital (5.6%) and the least infected was Omdurman Teaching Hospital (1.6%). According to hospital units surgery is the leading department which hosted 61.6% of the resistance genes, followed by the ICU (24%), then internal medicine (12%) and the least infected department was the pediatric unit (2.4%).

**Table 2:** extended spectrum β-lactamase (ESBL) genotypes among the major *Enterobacteriaceae* clinical isolates from patients in Khartoum, Sudan (2014-2015)

Genotype	*Escherichia coli*	*Klebsiella pneumoniae*	*Klebsiella oxytoca*	*Proteus mirabilis*	*Enterobacter cloacae*	Total
*bla*TEM	3	1	0	2	0	6
*bla*SHV	0	0	0	0	0	0
*bla*CTX-M	2	0	0	0	0	2
Total single ESBL	5	1	0	2	0	8
*bla*TEM + *bla*SHV	0	1	0	0	0	1
*bla*TEM + *bla*CTX-M	21	4	2	2	3	32
*bla*TEM + *blaSHV* + *bla* CTX-M	1	16	0	0	0	17
> 1 genes	22	21	2	2	3	50

## Discussion

The growing international passing through, communications and trade is causing an increase in the transfer ESBL and other antibiotic resistance mediating genes. Such increase in ESBL genes worldwide represents failures to treatment using the empirical approach. Many serious urinary, gastrointestinal and wounds infections fall within these categories, but are no longer treatable with beta-lactam antibiotics [6]. Data on existing levels of antimicrobial resistance in common pathogens is valuable in putting together proper choice of treatment [20]. Clinicians should be aware of the possible treatment failures in connection with infections caused by ESBL producing bacteria, particularly gram negative in the family *Enterobacteriaceae* [21]. Our data confirmed that the frequency of *bla*TEM (86%) and *blaCTX-M* gene (78%) were high among *Enterobacteriaceae* isolates from patients from Khartoum hospitals. As expected *E. coli* and *K. pneumonia* held the highest number of resistance individual genes as well as having the highest frequency of harboring more than one gene (22 and 21, respectively).

The trend of multidrug-resistant profile associated with the currently analyzed genes (*bla*TEM, *bla*HSV and *bla*CTX-M) is alarming. For that reason, setting up a routine screening of ESBL in clinical isolates to prevent dissemination of resistant isolates in health care settings is very important and needed more frequently. Previous data published from the region had confirmed this phenomenon and our findings are in agreement with published data. 53.3% of MDR *E. coli* were found resistant to >7 antimicrobial agents and ESBL was detected in 32.7% of them [11]. However, β-Lactamase was detected in all isolates and high. Levels of resistance was noticed among the 3^rd^ generation cephalosporin [12].Isolates capable of developing β-lactamases need to be recognized and dealt with appropriately. Such isolates are responsible for many nosocomial outbreaks and many fatalities and hospital costs due to treatment failure among infected patients, [22, 23]. Options in the treatment of ESBL-producing bacteria are very narrow. Carbapenems are the treatment of choice for dealing with crucial infections caused by ESBL-producing organisms. However, carbapenem-resistant isolates have lately been described [24].

*Enterobacteriaceae* are well adapted to exchange genetic stuff. Resistance is mainly owing to transposable resistance genes mediated in most by plasmids. Different mobile genetic elements are to be blamed for encapsulating these genes from chromosomes of different bacterial species and shift them “horizontally” between bacteria. Carriage of numerous resistance genes on one plasmid permits bacterial cell to attain multi-resistance in one move [25].

## Conclusion

ESBLs are considered to be one of the most important antibiotic resistance mechanisms and the degree of high resistance rates is coincide in this study with the prevalence of resistance genes. The highest frequency for ESBL production is in *E. coli* followed by *K. pneumonia* and also *K. oxytoca, P. mirabilis* and some other members of the *Enterobacteriaceae*. This study provides further evidence of the global dissemination of *bla*CTX-M and *bla*TEM and emphasizes the need for appropriate epidemiological monitoring. In this study, *E. coli* was found to be the most predominant MDR species and the prevalence of ESBL producing *E. coli* and *Klebsiella spp*. was higher. The majority of ESBL producing *E. coli* and *Klebsiella spp*. were resistant to antibiotics used for treatment of many infections. This clinical threat of increased ESBL prevalence is creating significant therapeutic problems prompting an immediate need to formulate strategic policy initiatives to reduce their prevalence. Imipenem and amikacin were the most effective antibiotics and could be the drug of choice for treatment of infections caused by ESBL strains notably *E. coli* but to a lesser extend against *Klebsiella* spp. and *Acinetobacter* spp. since they showed more resistance strains. Information on the levels of antimicrobial resistance among common pathogens is useful in making an appropriate choice of empiric therapy.

### What is known about this topic

- ESBLs are considered to be one of the most important antibiotic resistance mechanisms;
- Multidrug resistance is emerging in many Gram negative pathogens and is associated with severe nosocomial infections.

### What this study adds

- The emergence of multi-resistant ESBL-producing Enterobacteriaceae isolates is of major concern and highlights the need for further surveillance in the Khartoum and other states;
- In order to reduce the spread and transmission of these ESBL-producing strains, rapid diagnostic techniques to detect these strains, have to be implemented for the successful surveillance and for the implementation of the correct treatment of these strains in hospitals.
